# Prognostic value of global longitudinal strain in hypertrophic cardiomyopathy: A systematic review and meta‐analysis

**DOI:** 10.1002/clc.23928

**Published:** 2022-09-30

**Authors:** Ying Yang, Dong Wu, Hui Wang, Yanting Wang

**Affiliations:** ^1^ Department of Ultrasound China‐Japan Union Hospital of Jilin University Changchun Jilin China; ^2^ Department of Radiology The First Bethune Hospital of Jilin University Changchun Jilin China

**Keywords:** GLS, HCM, hypertrophic cardiomyopathy, speckle tracking, ultrasound

## Abstract

**Background:**

As previously reported, impairment of left ventricular global longitudinal strain (LVGLS) is associated with myocardial fibrosis, arrhythmias, and heart failure in hypertrophic cardiomyopathy (HCM) patients.

**Hypothesis:**

This study aimed to estimate the association between LVGLS measured by echocardiography and major adverse cardiovascular events (MACE) in patients with HCM.

**Methods:**

Pubmed, Embase, Scopus, and Cochrane Library databases were systematically searched for evaluating the difference of LVGLS between MACE and non‐MACE and the relevance of LVGLS and MACE in HCM patients, mean difference (MD), and pooled hazard ratios (HR) with 95% confidence interval (CI) were calculated. Publication bias was detected by funnel plots and Egger's test, and trim‐and‐fill analysis was employed when publication bias existed.

**Results:**

A total of 13 studies reporting 2441 HCM patients were included in this meta‐analysis. Absolute value of LVGLS was lower in the group of HCM with MACE (MD = 2.74, 95% CI: 2.50–2.99, *p* < .001; *I* ^2^ = 0, *p* = .48). In the pooled unadjusted model, LVGLS was related to MACE (HR = 1.14, 95% CI: 1.06–1.22, *p* < .05, *I* ^2^ = 58.4%, *p* < .01) and there is a mild heterogeneity, and sensitivity analysis showed stable results. In the pooled adjusted model, LVGLS was related to MACE (HR = 1.12, 95% CI: 1.08–1.16, *p* < .05; *I* ^2^ = 0%, *p* = .442). Egger's tests showed publication bias, and trim‐and‐fill analysis was applied, with final results similar to the previous and still statistically significant.

**Conclusion:**

The meta‐analysis suggested that impaired LVGLS was associated with poor prognosis in HCM patients.

## 1 INTRODUCTION

Hypertrophic cardiomyopathy (HCM) is a common hereditary cardiovascular disease with an incidence of about 1 in 500 patients, and it is the most common cause of sudden death in young people.^1^ Its histopathology reveals hypertrophy of cardiomyocytes, disorganization of myocardial fiber bundles, hyperplasia of myocardial interstitial fibers, thickening of the walls of small coronary arteries between cardiomyocytes, and compression of the lumen, resulting in subendocardial myocardial ischemia, leading to abnormal systolic and diastolic function and microcirculatory disorders.^2^, ^3^ Traditionally, the treatment of HCM patients has focused on identifying the high risk of sudden cardiac death (SCD) and referring them for primary prevention implantable cardioverter defibrillators (ICD) or septal resection or alcoholic ablation.^1^, ^4^ Early diagnosis and appropriate prognostic stratification will reduce relevant complications and mortality by facilitating timely treatment.

Echocardiography is an important tool in the diagnosis and follow‐up of HCM. The left ventricular ejection fraction (LVEF) is one of the most commonly used cardiac function parameters in clinical practice. However, in HCM patients, the thickening of the ventricular wall indirectly causes the narrowing of the heart cavity, so that LVEF can remain within the normal range despite impaired left ventricular systolic function.^5^, ^6^ Therefore, myocardial deformation can bring additional prognostic information.^7^ 2‐dimensional speckle tracking (2D‐STE) is a modern technique regarding 2D transthoracic echocardiography using grayscale imaging, which allows spatial and temporal tracking of LV myocardial deformation.^5^, ^8^ Left ventricular global longitudinal strain (LVGLS) accurately quantifies the local myocardial deformability and is a powerful indicator of early systolic dysfunction, even before a significant reduction in LVEF. Currently, a growing number of scholars believe that 2D‐STE derived LVGLS may be an important indicator of the prognosis and the risk stratification of patients with HCM, but there is a lack of large sample size studies, so we sought to perform a meta‐analysis to investigate whether LVGLS was related to the prognosis of HCM.

## 2 METHOD

### 2.1 Literature research

A systematic search of Pubmed, Embase, Scopus, and Cochrane Library databases was conducted until May 1, 2022. The search terms were as follows: echocardiography, ultrasound, global longitudinal strain, GLS, longitudinal strain, strain, speckle tracking, and HCM.

### 2.2 Eligibility criteria and study selection

Inclusion criteria: (1) HCM diagnostic criteria: The largest left ventricular wall thickness ≥15 mm, and increased load‐induced myocardial thickening such as hypertension, aortic stenosis, or congenital subaortic septum needs to be excluded^1^; (2) follow‐up period of at least half a year; (3) LVGLS was evaluated by 2D‐STE; (4) sufficient data to retrieve hazard ratios (HR) with 95% confidence interval (CI) obtained by Cox regression analyses; (5) all articles included were approved by the Research Ethics Committee.

Exclusion criteria: (1) review, case reports, experiments on animals, comments, editorials, conference abstract, duplicate publications; (2) age <16; (3) the number of cases is less than 10; (3) non–English language literature.

### 2.3 Data extraction and quality assessment

Basic information for the included articles including author, publication years sample size, study design, age, gender, LVEF, and LVGLS. Means and standard deviations of LVGLS in the MACE and non‐MACE groups, as well as the unadjusted and adjusted HR with 95% CI, were extracted from the included studies. Newcastle–Ottawa Scale (NOS) was adopted to evaluate the quality of the included publications. All data were extracted independently by two authors.

### 2.4 End‐point

The main outcome was MACE, including composite death and any cardiovascular events (heart failure, ventricular tachyarrhythmia, and ICD implantation). Heart failure was defined as NYHA class III or IV with new or worsening symptoms of heart failure, including hospitalization for heart failure.

### 2.5 Statistic analysis

The effect estimates were reported as mean difference (MD) of LVGLS among MACE and non‐MACE groups and HR with 95% CI, which was pooled from unadjusted and adjusted model effect estimates from the included studies, and with *p* < .05 considered to be statistically significant.

The *I*‐squared (*I* ^2^) statistic and Cochrane Q‐statistic were used to evaluate heterogeneity among included studies, *I* ^2^ > 50% or *p* < .05 indicated the existence of heterogeneity, and a random‐effect model would be applied. Sensitivity analysis would be employed additionally.

Publication bias was detected by funnel plots and Egger's test. We concluded that there was no publication bias when the included literature was evenly and symmetrically distributed on both sides of the vertical axis of the funnel plots, and Egger's test showed that there was no publication bias when *p* > .05. The trim‐and‐fill analysis was employed when publication bias existed. All statistical analyses were performed by Stata 15.1 software.

## 3 RESULT

**Figure 1:**
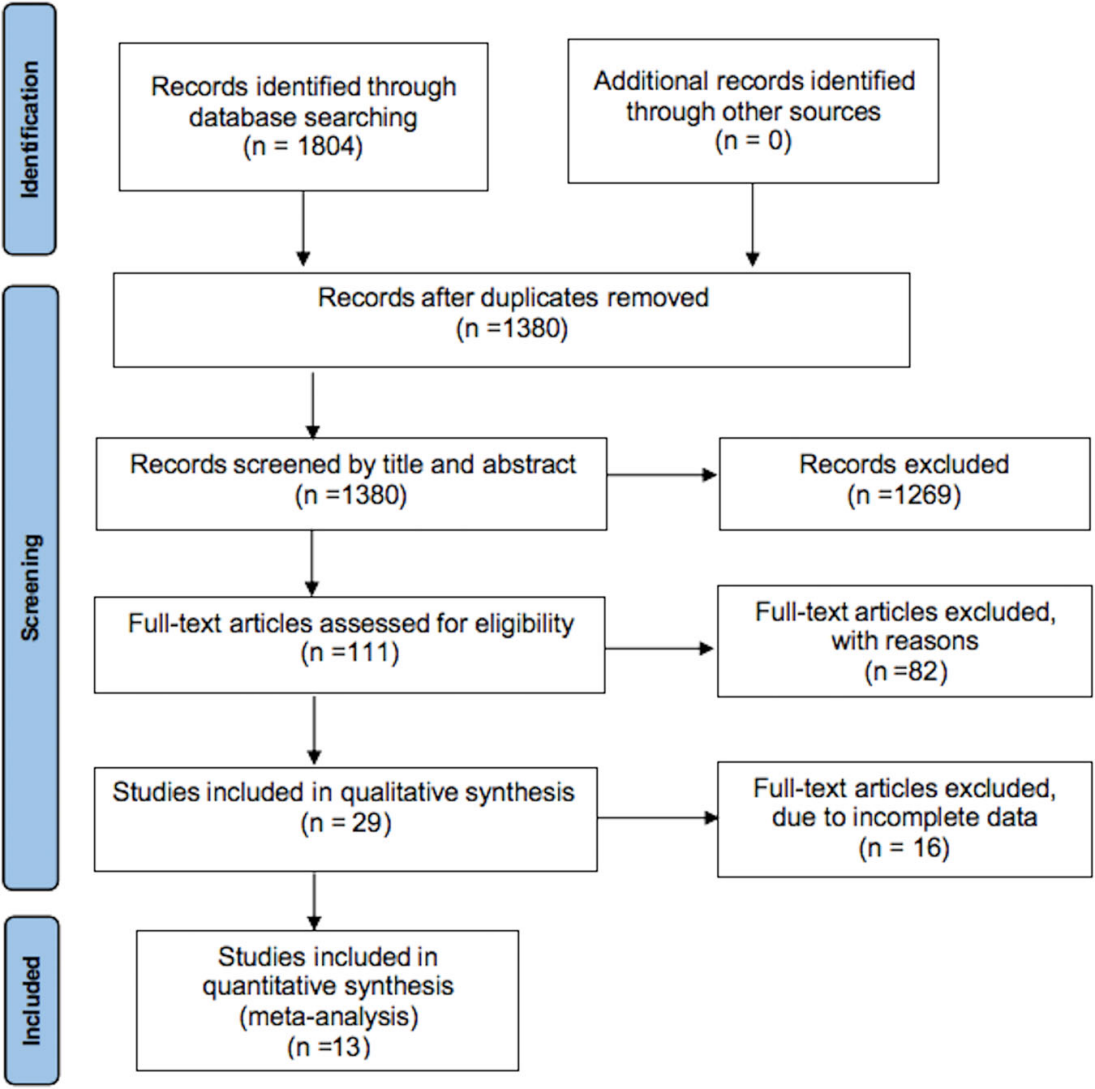
Flow diagram

After carefully reading the full text, a total of 13 studies reporting 2441 HCM patients were included in this meta‐analysis,^9^, ^10^, ^11^, ^12^, ^13^, ^14^, ^15^, ^16^, ^17^, ^18^, ^19^, ^20^, ^21^ the flow diagram is shown in Figure 1. The following features of eligible studies and risk of bias assessment using the NOS are summarized in Table 1.

**Table 1:** Summary of patient characteristics

Author	Year	No.	Males (%)	Mean age	Country	Design	Mean or median of follow‐up	LV‐GLS (%）	LVEF (%)	Platform (vendor)	NOS
Paraskevaidis^9^	2009	50	58	51 ± 18	Greece	Prospective	12 months	−14 ± 4	71 ± 7	GE Vivid 7	9
Debonnaire^10^	2014	92	69	50 ± 14	Netherlands	Retrospective	4.7 years	−13.3 ± 3.5	70 (64, 76)	GE Vivid 5, Vivid7, E9, GE‐Vingmed, Milwaukee, WI	8
Hartlage^11^	2015	79	54	44 ± 16	US	Retrospective	22 months	−14.3 ± 4.2	63 ± 6	GE Vivid 7 and E9	8
Reant^12^	2015	115	66	51.9 ± 15.2	France	Prospective	19 ± 11 months	16.5 ± 3.6	71.0 ± 6.9	GE Vivid 9	8
Candan^13^	2017	63	77.8	48.5 ± 14.2	Turkey	Retrospective	21.5 months	−12.1 ± 3.4	‐	GE Vivid 7	8
Liu^14^	2017	400	66	51 ± 15	US	Prospective	3.1 years	−16 ± 4	65 ± 8	GE Vivid 7/Vivid E9	6
Ozawa^15^	2017	41	65.9	60 ± 13	Japan	Retrospective	32.1 months	‐	‐	Philips IE33	7
Hiemstra^16^	2017	427	66	52 ± 15	Netherlands	Retrospective	6.7 years	−15 ± 4	65 ± 9	GE Vivid 5, Vivid 7, E9, GE‐Vingmed, Milwaukee, WI	7
Hiemstra^17^	2018	236	68	50 ± 14	Netherlands	Retrospective	6.5 years	−16 ± 4	65 ± 8	GE Vivid 5, Vivid 7, E9, GE‐Vingmed, Milwaukee, WI	7
Vergé^18^	2018	179	‐	‐	France	Prospective	2.8 ± 1.5 years	‐	‐	GE Vivid 7/E9	6
Essayagh^19^	2021	307	65.8	54 ± 17	France	Retrospective	21 months	−15 ± 4	67 ± 10	GE	7
Zegkos^20^	2021	250	67.2	50.8 ± 15.9	Greece	Prospective	2.5 ± 1.2 years	−15 ± 4	69.2 ± 9	GE Vivid S70	8
Candan^21^	2022	202	68	48 ± 13.9	Turkey	Retrospective	45.9 months	−13 (−15,−11)	63.4 ± 6.3	GE Vivid 7	8

Abbreviations: LVEF, left ventricular ejection fraction; LVGLS, left ventricular global longitudinal strain; NOS, Newcastle–Ottawa Scale

### 3.1 MD between MACE and non‐MACE in HCM patients

**Figure 2:**
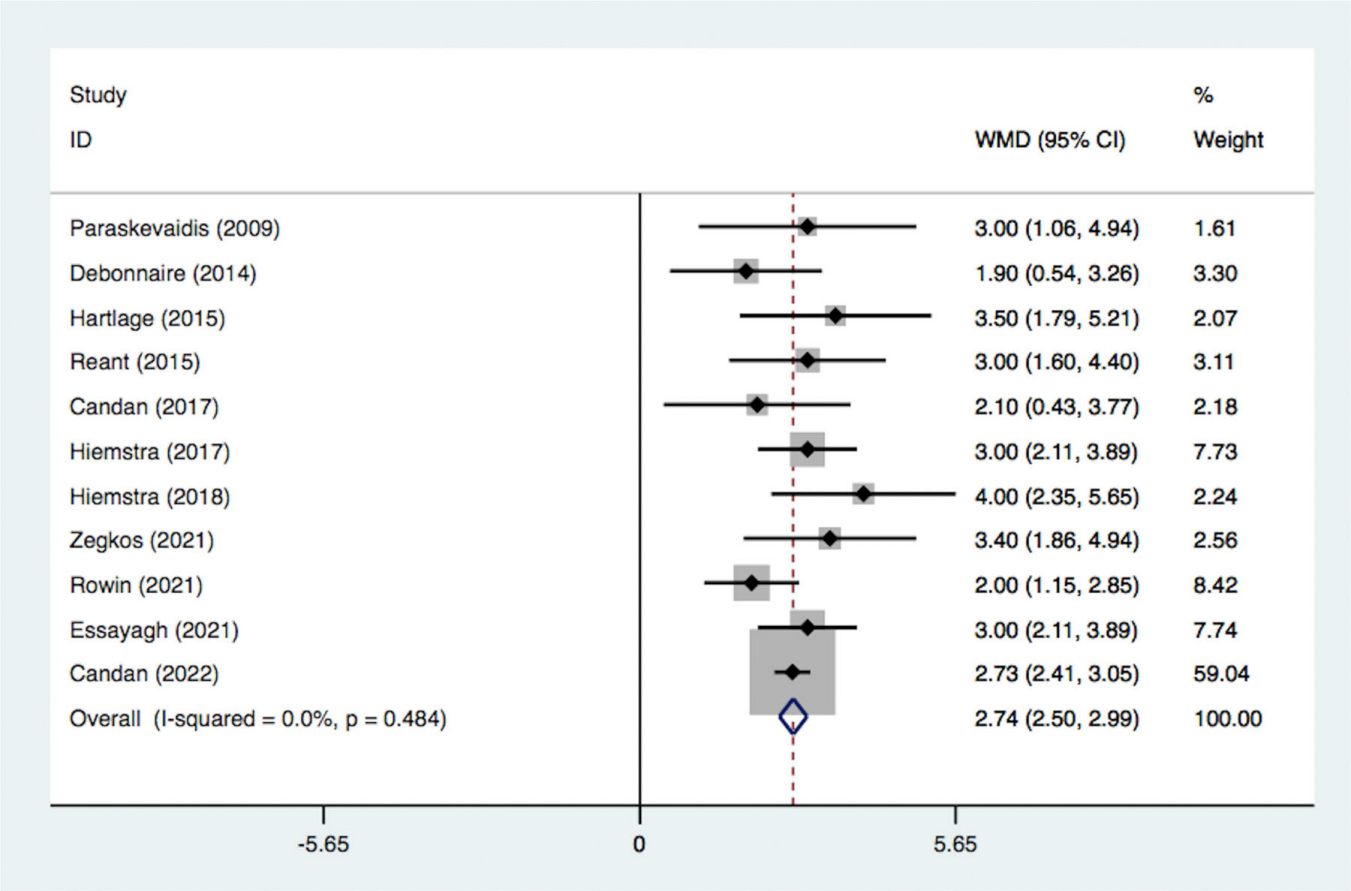
Mean difference in LVGLS between MACE and non‐MACE. CI, confidence interval; LVGLS, left ventricular global longitudinal strain; MACE, major adverse cardiovascular events.

This set of analyses included 11 articles in which we extracted and combined the mean and standard deviation of LVGLS in MACE and non‐MACE groups from the included articles. Absolute value of LVGLS was lower in the group of HCM with MACE (MD = 2.74, 95% CI: 2.50–2.99, *p* < .001; *I* ^2^ = 0, *p* = .48), as is shown in Figure 2.

### 3.2 Correlation between LVGLS and MACE

**Figure 3:**
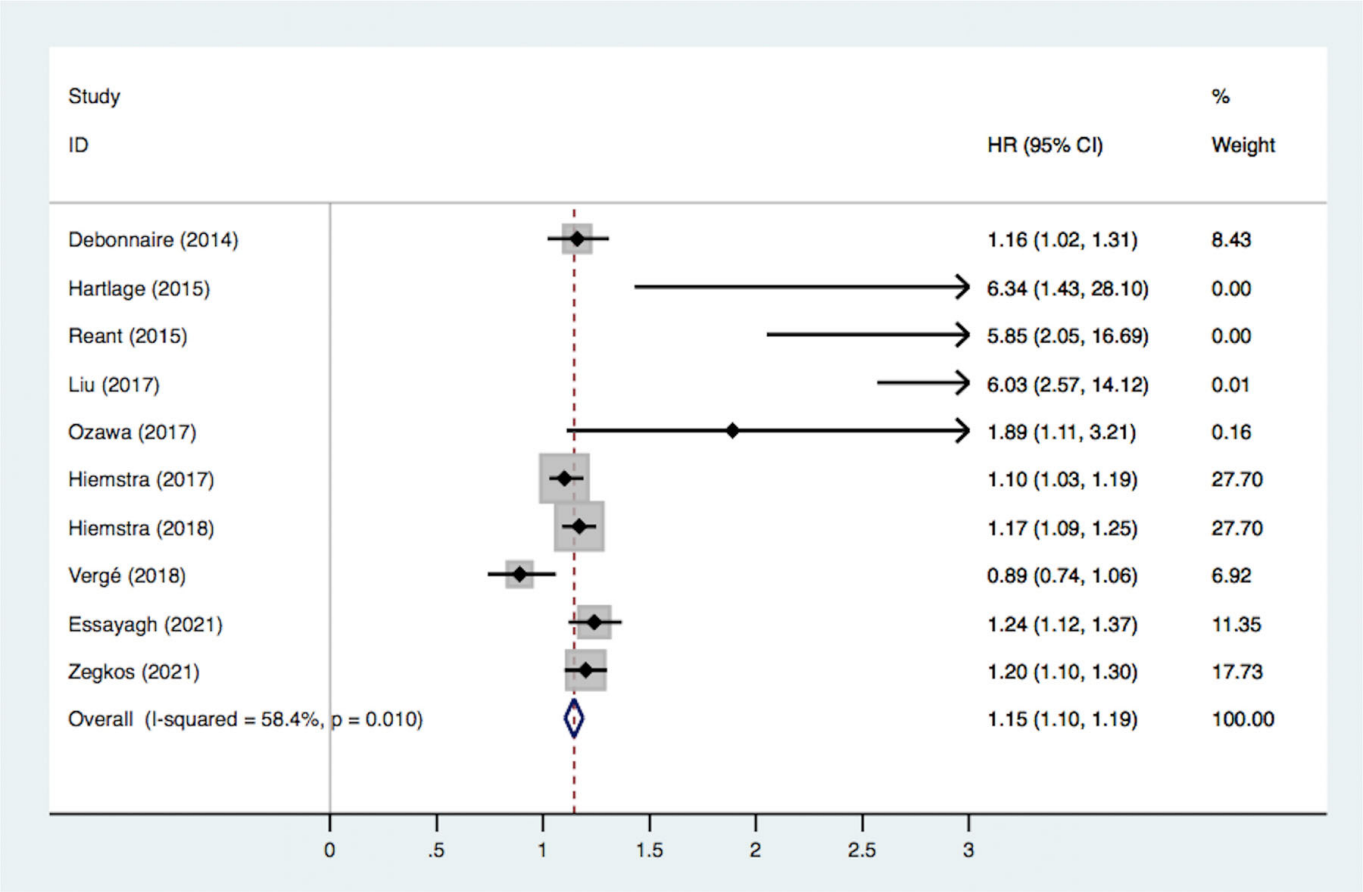
Association between LVGLS and MACE (unadjusted model). CI, confidence interval; HR, hazard ratios; LVGLS, left ventricular global longitudinal strain; MACE, major adverse cardiovascular events.

**Figure 4:**
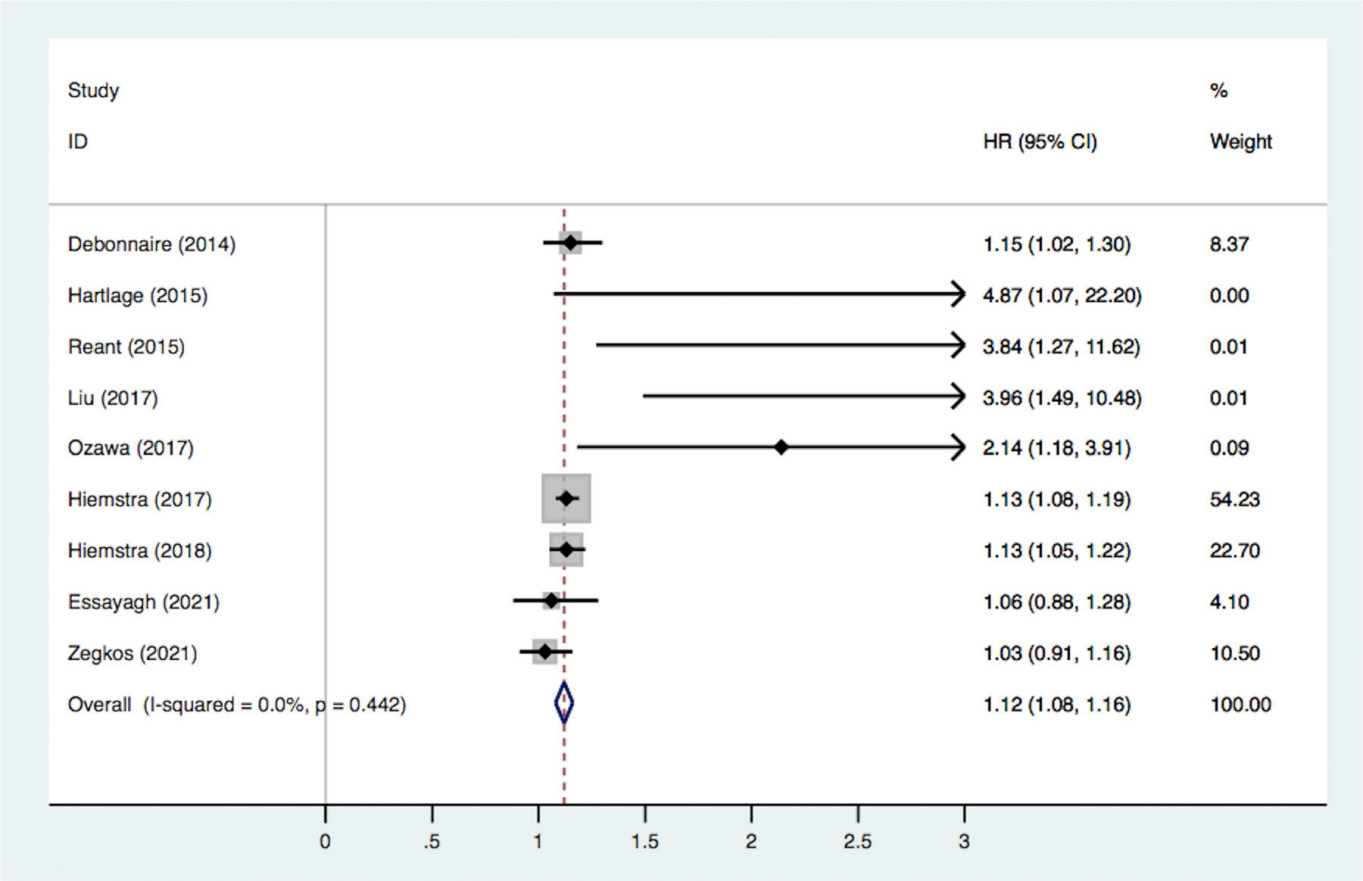
Association between LVGLS and MACE (adjusted model). CI, confidence interval; HR, hazard ratios; LVGLS, left ventricular global longitudinal strain; MACE, major adverse cardiovascular events.

Ten articles reported the correlation between LVGLS and MACE, of which nine articles used multivariate regression analysis to identify independent predictors of the endpoint, we analyzed the unadjusted and adjusted models respectively. There was mild heterogeneity in the pooled unadjusted model (*I* ^2^ = 58.4%, *p* < .01), and therefore a random‐effects model was adopted, showing that impaired LVGLS significantly increased the risk of MACE (HR = 1.14, 95% CI: 1.06–1.22, *p* < .05) (Figure 3). In the pooled adjustment model, impaired LVGLS significantly increased risk of MACE (HR = 1.12, 95% CI: 1.08–1.16, *p* < .05), with no significant heterogeneity (*I* ^2^ = 0%, *p* = .442) (Figure 4).

### 3.3 Sensitivity analysis

The sensitivity analysis of the unadjusted model was conducted by eliminating articles one by one, the results ranged from (HR = 1.12, 95% CI: 1.03–1.22) to (HR = 1.17, 95% CI: 1.11–1.23), and found that heterogeneity (*I* ^2^ = 28.0, *p* = .185) was significantly reduced after removing the article of Vergé et al. (Supporting Information: Figure 1).

## 4 PUBLICATION BIAS

In the first part of the analysis, the funnel plot was symmetrical as shown in Supporting Information: Figure 2A, and there was no publication bias demonstrated by Egger's test (*p* = .273).

In the second part of the analysis, the funnel plots of the unadjusted and adjusted models were both asymmetric (Supporting Information: Figure 2B,C), we conducted Egger's tests separately, which indeed suggested publication bias (*p* = .033, 0.025) (Supporting Information: Figure 3), and trim‐and‐fill analyses showed three theoretically missing studies, respectively (Supporting Information: Figure 4). After filing, pooled HR = 1.156, 95% CI: 1.015–1.315, *p* = .028 (unadjusted model), pooled HR = 1.122, 95% CI: 1.083–1.164, *p* < .001 (adjusted model).

## 5 DISCUSSION

A total of 13 studies with 2441 patients were included in this study. The first set of analyses showed that LVGLS in HCM patients with MACE was significantly impaired. In the second part, we analyzed the correlation between LVGLS and MACE, and the unadjusted model included 10 articles with mild heterogeneity, therefore the random‐effects model was used, and then sensitivity analysis was performed by excluding articles one by one, finding that the heterogeneity was reduced after removing the article by Vergé et al.^18^ We considered it to be the source of the main heterogeneity, but after reading through the whole article we were still unable to identify the key factors contributing to the overall heterogeneity. The adjusted model included nine articles that used multivariate regression analysis to identify independent predictors of endpoints, and the combined results showed a significant correlation between impaired LVGLS and MACE, concluding that LVGLS was an independent predictor of MACE.

It is well known that LVGLS is superior to LVEF in detecting early left ventricular systolic dysfunction, so the application of LVGLS in the clinical practice of HCM has been a research hotspot. The studies of Haland and colleagues,^22^, ^23^ which were not included in this meta‐analysis due to the years of follow‐up, both performed 24–48 h electrocardiogram (Holter) and showed that LVGLS was significantly reduced in HCM patients with ventricular arrhythmias (VAs), and indicated that global or septal longitudinal strain was associated with the development of VAs. Cardiomyocyte hypertrophy and myocardial interstitial fibrosis in HCM are important factors in the development of VAs, and it is worth mentioning here that some studies have shown that impairment of LVGLS correlates with the degree of myocardial fibrosis, myocardial disorder and fibrosis may be the main determinants of abnormal myocardial deformation in HCM patients.^24^, ^25^, ^26^ The study of Cui et al.^27^ followed up patients with HCM who underwent septal myectomy and showed that postoperative LVGLS could still be used as an indicator to evaluate prognosis, with lower survival rates in the group with poorer LVGLS. Impaired LVGLS is related to an increased risk of VAs and appropriate ICD therapies.^28^ In the study of Saito et al.,^26^ all HCM patients had normal LVEF, but during the follow‐up period, five patients had cardiac events, and these five patients all occurred in the group with poor LVGLS. These findings suggest that LVGLS reduction is associated with myocardial fibrosis and poor prognosis in patients with HCM and is considered as a potential marker of increased risk, which can be used as a prognostic indicator. However, these studies were all small samples and single center, so we conducted this meta‐analysis to confirm that, although there was mild heterogeneity, the sensitivity analysis confirmed that our study results were stable and reliable.

Publication bias tests showed that although publication bias was present in the second set of analyses in both adjusted and unadjusted models, and further trim‐and‐fill analysis considered possible missing articles, the final results remained significant after filling, suggesting that publication bias had little effect on our results. Considering these findings, we believe that impaired LVGLS plays a strong prognostic role in HCM patients.

In addition, it has been shown that longitudinal strain is highly reproducible and outperforms circumferential and radial strain in detecting early myocardial dysfunction.^29^, ^30^ The value of LVGLS has also been demonstrated in the long‐term prognostic evaluation of cardiac diseases such as ischemic cardiomyopathy, heart failure, and aortic stenosis.^31^, ^32^, ^33^

In conclusion, 2D‐STE not only detects LV systolic dysfunction at an early stage but also plays an important role in predicting poor prognosis in patients with HCM. This finding may contribute to better risk stratification of patients with HCM and has considerable clinical value.

## 6 LIMITATIONS

This meta‐analysis has several limitations. First, the predictors used in the multivariate adjustment varied across studies. Second, a wide variety of endpoints were included. Finally, in the included studies, we did not perform subgroup analyses due to insufficient information.

## 7 CONCLUSION

LVGLS measured by 2D‐STE is a powerful and independent predictor of adverse events in patients with HCM.

## CONFLICT OF INTEREST

The authors declare no conflict of interest.

## Supporting information

Supporting information.

## Data Availability

The data that support the findings of this study are available from the corresponding author upon reasonable request.
