# Supplementary material for: Prognostic value of global longitudinal strain in hypertrophic cardiomyopathy: A systematic review and meta‐analysis
Source: Clin Cardiol. 2022 Sep 30;45(12):1184–91. doi: 10.1002/clc.23928 (PMC9748764; doi:10.1002/clc.23928)
Supplement: Supplementary file 1 — Supporting information. [file CLC-45-1184-s001.docx]

**Supplemental figures**

**
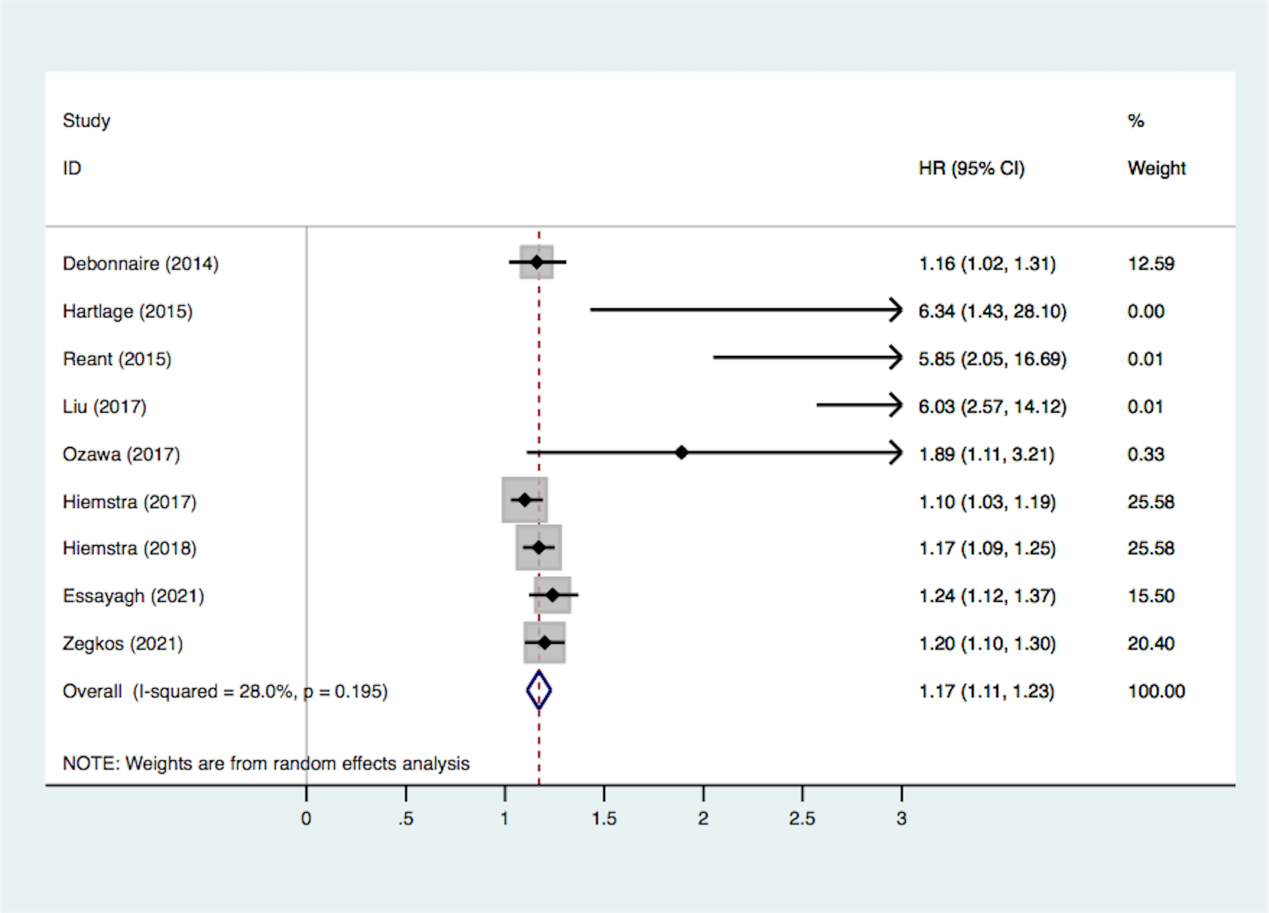
**

SFigure 1. Sensitivity analysis (exclude the article of Vergé et al. )


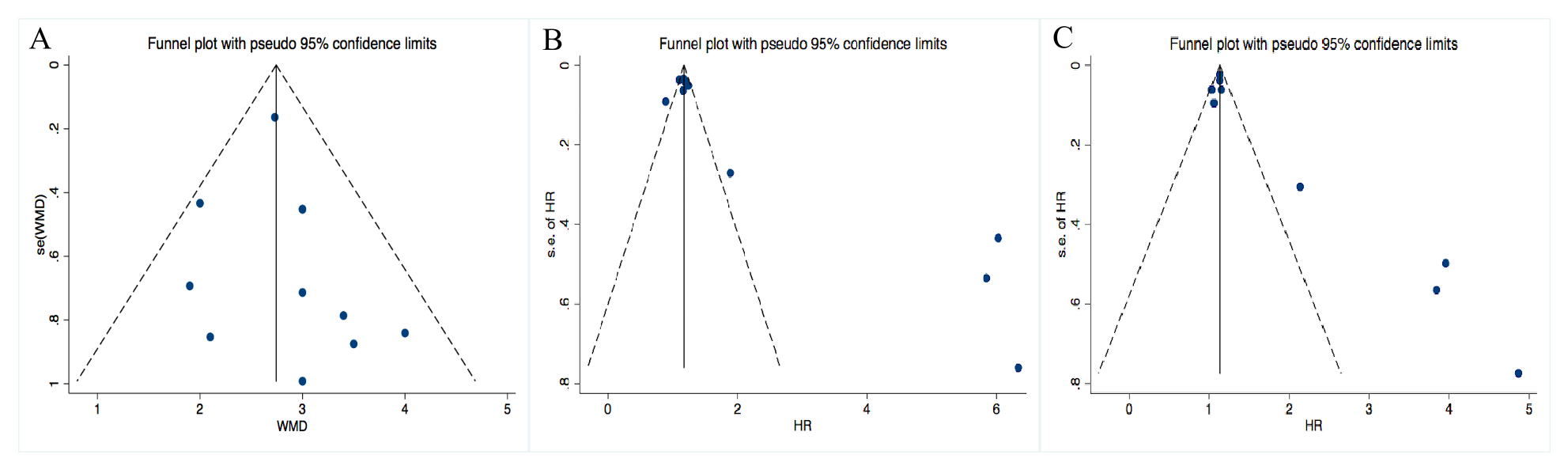


SFigure 2. Funnel plot analysis：comparison of LVGLS between MACE and non-MACE groups (A), correlation between LVGLS and MACE (unadjusted model) (B), and correlation between LVGLS and MACE (adjusted model) (C)


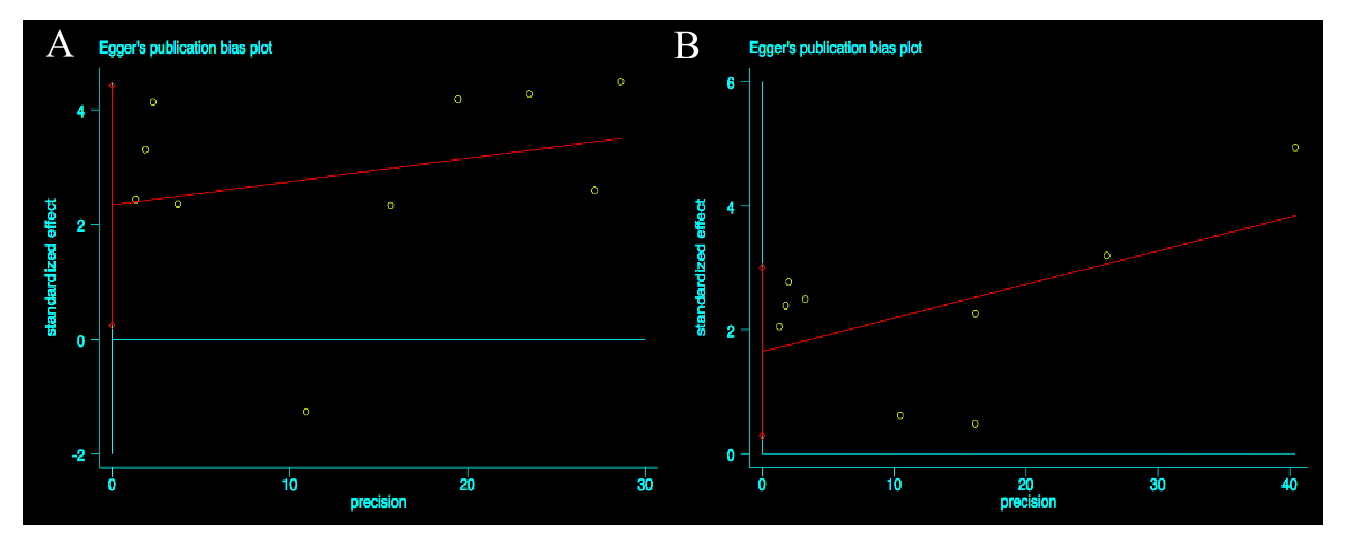


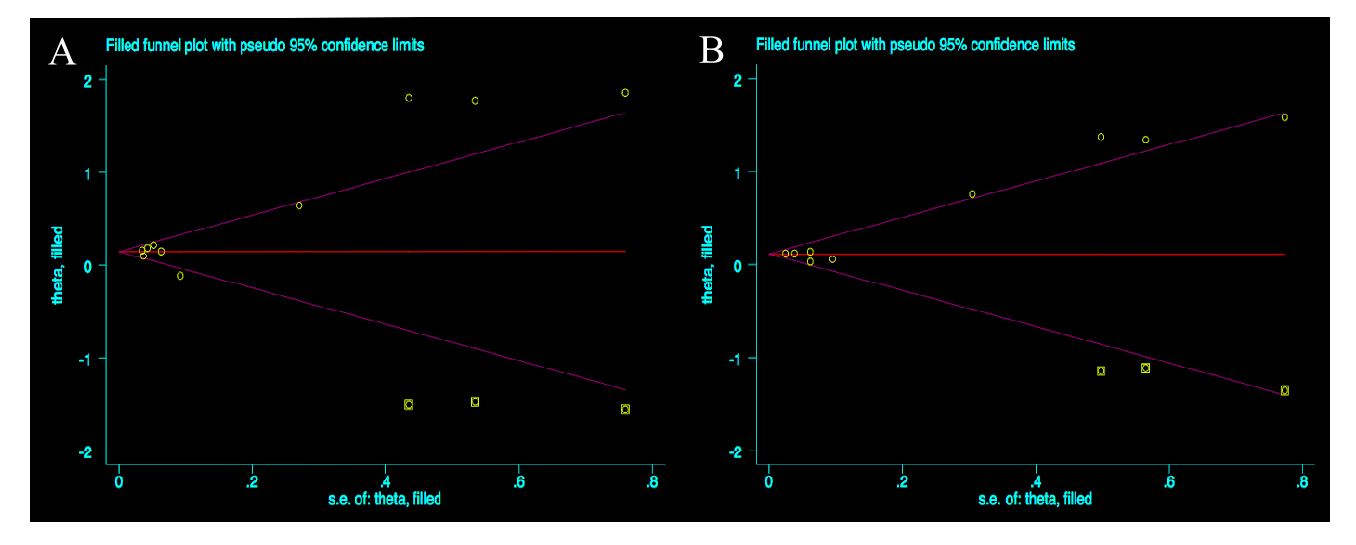
SFigure 3 Egger’s test: unadjusted model (A) and adjusted model (B)

SFigure 4 Trim and fill analysis : unadjusted model (A) and adjusted model (B)
